# Research progress on the mechanism of radiation enteritis

**DOI:** 10.3389/fonc.2022.888962

**Published:** 2022-09-05

**Authors:** Jinjia Fan, Binwei Lin, Mi Fan, Tintin Niu, Feng Gao, Bangxian Tan, Xiaobo Du

**Affiliations:** ^1^ Departmant of Oncology, National Health Commission Key Laboratory of Nuclear Technology Medical Transformation (Mianyang Central Hospital), Mianyang Central Hospital, School of Medicine, University of Electronic Science and Technology, Mianyang, China; ^2^ Department of Oncology, Affiliated Hospital of North Sichuan Medical College, Nan Chong, China

**Keywords:** Radiation enteritis, mechanism, intestinal epithelial cells, intestinal stem cells, vascular endothelial cell, intestinal microflora

## Abstract

Radiation enteritis (Re) is one of the most common complications of radiation therapy for abdominal tumors. The efficacy of cancer treatment by radiation is often limited by the side effects of Re. Re can be acute or chronic. Treatment of acute Re is essentially symptomatic. However, chronic Re usually requires surgical procedures. The underlying mechanisms of Re are complex and have not yet been elucidated. The purpose of this review is to provide an overview of the pathogenesis of Re. We reviewed the role of intestinal epithelial cells, intestinal stem cells (ISCs), vascular endothelial cells (ECs), intestinal microflora, and other mediators of Re, noting that a better understanding of the pathogenesis of Re may lead to better treatment modalities.

## Introduction

The intestine is particularly sensitive to ionizing radiation (IR).Vomiting, diarrhea, abdominal pain, bleeding, obstruction, perforation, and nutrient absorption disorder are common radiation toxicities in the intestine, which may lead to the decline of the patients’ quality of life and even death (1). Several therapy-related factors such as single-fraction dose, total dose, irradiated volume of the intestine, and the use of concurrent chemotherapy or biotherapy will influence the incidence and severity of intestinal radiation toxicity (2, 3). Moreover, patient-related factors like previous abdominal surgery, inflammatory bowel disease, diabetes, and vascular disorders may also affect the occurrence of Re (3). To solve the complications caused by radiotherapy, many strategies have been developed to relieve symptoms, including limiting intestinal irradiation dose and using a lower fractionated dose. However, these compromises may reduce the anti-tumor effect (3, 4). An in-depth study of the mechanism of Re is very important for finding new and effective strategies to prevent and treat Re. This review mainly summarized the current research mechanism and intervention measures related to intestinal injury caused by radiation therapy.

### Structure and function of the intestinal barrier

**Figure 1:**
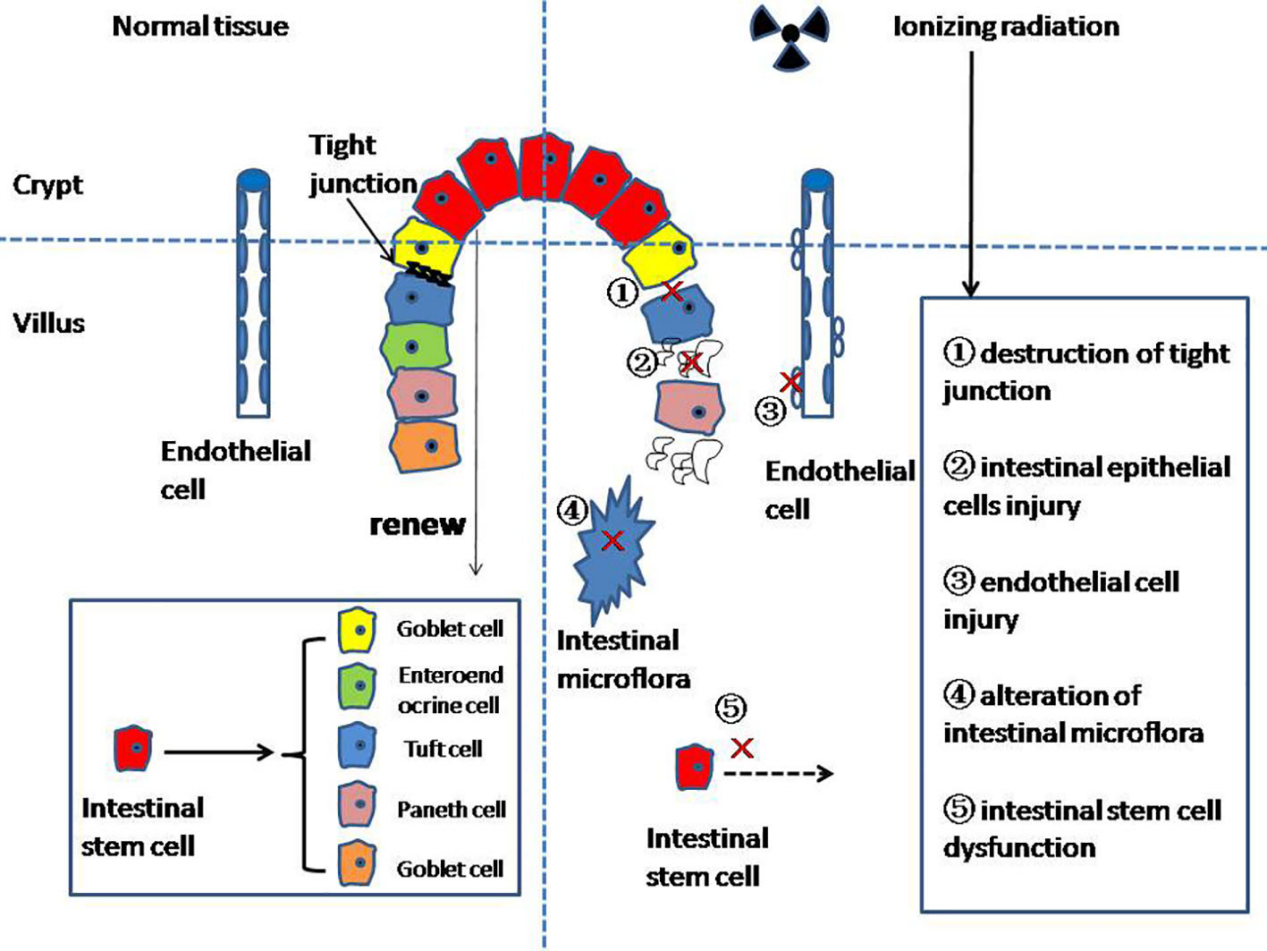
The mechanism of radiation enteritis.

The function of the intestinal barrier is for the absorption of nutrients through the mucosa and the prevention of injury from other toxic substances (5). The mucous layer is the first physical line of defense of the intestinal barrier, preventing bacteria and viruses from directly contacting epithelial cells (6). The main component of the mucous layer is highly glycosylated mucus, forming a gelatinous sieve structure outside the intestinal epithelium. Mucin 2 (MUC2) secreted by goblet cells is the most abundant mucine in the mucous layer, and the expression of MUC2 is closely related to the occurrence of enteritis, but the specific mechanism is still unclear (7, 8). The tight junctions (TJs) between the intestinal epithelial cells (IECs) constitute the second barrier of the intestinal barrier (9, 10), which are the determinant of the intestinal barrier function (5). The TJs’ structure is composed of transmembrane proteins such as claudin, occludin, tricellulin, and junction adhesion molecules (11, 12). TJs connect IECs to form a continuous polarized single-layer structure, separating the lumen from the lamina propria (13, 14). The lamina propria forms the last layer of the intestinal barrier, which is composed of immune cells, endothelial cells (ECs), myofibroblasts, matrix components, etc. In addition, the intestinal microbiota is also involved in the formation of the intestinal barrier (15). It should be noted that the intestinal barrier is not a static structure; it is always in dynamic equilibrium (**Figure 1**).

At present, studies on the mechanism of Re focus on the following aspects: the destruction of the intestinal epithelium, intestinal stem cell (ISC) injury, intestinal microvascular changes, and intestinal microflora disruption, among others.

## Intestinal epithelium injury

Radiotherapy can lead to an increase in intestinal epithelium barrier and permeability, which is closely related to the destruction of the TJs’ structure by IR. Morini et al. found that the expression of occludin, claudin, ZO-1, and ZO-2 was related to Re. They believed that when Re occurred, ZO-1, claudin-1, and occludin in TJs would recombine or break, resulting in the destruction of the intestinal barrier (16).

The adherent junctions (AJs) located below TJs are multiprotein complexes that indirectly regulate the TJs’ maturation and integrity. Gupta et al. (17) found that the AJs’ structure between IECs of mice disintegrated, expanded, and ruptured after exposure to IR. The amino acid-based oral rehydration solution (AA-ORS), including threonine, valine, serine, tyrosine, and aspartic acid, reduced dilation within AJs and reversed radiation-induced functional and structural disruption of the intestinal barrier (17).

Autophagy plays an important role in maintaining intestinal barrier homeostasis and regulates the apoptosis and necrosis of IECs (18). It has been proven that intracellular mitochondria produce a large number of intracellular reactive oxygen species (ROS), and when autophagy is insufficient, ROS levels will increase (19). ROS can cause structural damage and dysfunction in DNA. Ionizing radiation, on the other hand, inhibits autophagy, leading to damage to the intestinal barrier (20).

Resveratrol is a polyphenol synthesized in grape leaves and grape skins (21). Qin et al. (22) reported that the apoptosis level of IECs in the resveratrol-pretreated group was significantly lower than that in the irradiation group alone 24 h after exposure to ionizing radiation. Resveratrol can promote autophagy by activating the SIRT1 pathway to protect IECs and prevent the occurrence of Re (22).

## Intestinal stem cell injury

IECs can renew themselves rapidly every 4–5 days (23). The ability to renew itself in the long-term can only be maintained by intestinal stem cells (ISCs) in the crypt (24). ISCs mainly form two differentiated epithelial lineages: (1) the enterocyte lineage, and (2) the secretory lineage. The enterocyte lineage is mainly responsible for absorbing nutrients. The secretory lineage consists of Paneth cells, which regulate the maintenance and differentiation of Lgr5+ ISCs; the mucus-secreting goblet cells; enteroendocrine cell (EEC); and tuft cells (**Figure 1**).

The main cause of Re is ISC death, which leads to the loss of key cells and destruction of crypt structures (25, 26).

Thiazolidine hydrochloride (TCZC01) is a novel compound synthesized by Zingerone. This paper concludes that pretreatment with TZC01 can significantly improve intestinal crypt apoptosis, increase the number of Lgr5+ ISCs, and even reduce intestinal cell apoptosis, thus protecting the intestinal barrier from IR damage. However, the mechanism of TZC01 is not explained in this paper (27).

The ISCs’ niche is not a constant, but a complex and dynamic environment. The stem cell zone is surrounded by enteric neurons, endothelial cells (ECs), smooth muscle cells (SMCs), intraepithelial lymphocytes, macrophages, and fibroblasts/myofibroblasts together with the extracellular matrix (ECM). Wnt, Notch, bone morphogenetic protein (BMP), and Hedgehog are the major signaling pathways involved in the maintenance of the ISCs’ niche (28).

### Wnt/β-catenin pathway

The Wnt/β-catenin pathway plays an important regulatory role in intestinal tissue homeostasis (29). The stability of the environment within the intestinal tissue is coordinated and controlled by the self-renewal, regeneration, and reprogramming of stem cells (30). The typical Wnt signaling pathway is mediated by β-catenin, and β-catenin increases rapidly when Wnt ligands bind. After β-catenin enters the nucleus, it acts as a transcription co-activator of transcription factor 4 (TCF4), leading to the transcription and expression of a series of genes (31). A previous study showed that the Wnt/β-catenin signaling pathway played an important role in the self-renewal and proliferation of ISCs after radiation injury (32). However, we still do not know how the Wnt signal is transmitted to the target cell as the intercellular signal.

As reported by Li et al., epicatechin can salvage the ISCs’ activity and activate the Wnt/β-catenin pathway to induce crypt regeneration (33).

Podophyllotoxin combined with rutin (G-003M) is considered to reduce intestinal damage caused by radiation, and the main mechanism may enhance β-catenin nuclear translocation-promoted Lgr5 (+) ISC renewal through the Wnt/β-catenin signaling pathway (34).

Pretreatment with heat-killed *Salmonella typhimurium* (HKST) upregulated the nuclear localization of ß-catenin through the Wnt/b-catenin pathway. Moreover, pretreatment with HKST greatly increased the value of intestinal cells, significantly improved the structure and function of crypts, and reduced intestinal damage caused by ionizing radiation to prevent radiation enteritis (35).

### Notch pathway

The Notch pathway is one of the key signaling pathways that maintain the balance of intestinal epithelial cell proliferation and differentiation. The Notch signaling pathway relies on cell-to-cell signaling, in which a cell provides a Notch ligand to adjacent cells expressing the Notch receptor (36). However, it usually results in the opposite fate of neighboring cells (lateral inhibition) (37). When the Notch receptor and ligand binding are activated, the Notch intracellular domain (NCID) is hydrolyzed and released into the nucleus and changes the gene expression in coordination with transcription factors, especially recombination signal binding protein J (RBP-J) (19).

Ghrelin is a hormone mainly produced by gastrointestinal endocrine cells (38). Recently, Kwak et al. (39) found that ghrelin could retain the proliferative function of IECs after irradiation by activating the Notch pathway *in vitro*. Further *in vivo* experiments confirmed that ghrelin could alleviate acute intestinal injury caused by radiation. The authors speculated that ghrelin is a potential strategy for the treatment of Re by activating the Notch pathway to retain the proliferative ability of IECs and repair intestinal barrier injury.

Park et al. (25) found that Valproic acid (VPA) was an effective intestinal protective agent. The results showed that IR reduced the activity of intestinal organoid by 70%, while pretreatment with VPA only reduced the activity of intestinal organoid by 30%. Further studies demonstrated that VPA significantly upregulated NOTCH1 mRNA level, activated the Notch pathway to reduce IR damage to LGR5+ cells, and improved crypt regeneration.

### Bone morphogenetic protein signaling pathway

The BMP is the most important part of the transforming growth factor β (TGFβ) superfamily (40). BMP binds to the complex on the cell membrane (composed of serine/threonine kinase) and activates the intracellular heteromeric Smad complex, thereby regulating gene expression (41–43). On the other hand, the BMP signaling pathway has been shown to negatively regulate the self-renewal of Lgr5+ ISCs by inhibiting Wnt signaling (42, 44, 45). The BMP signaling pathway is negatively regulated by Gremlin, which is secreted by trophoblast cells (46, 47).

Martín-Alonso et al. (48)found that radiation-induced intestinal epithelial injury could not be repaired without the membrane-bound matrix metalloproteinase-17 (MMP17) expressed by smooth muscle cells. MMP17, an antagonist of the BMP signaling pathway, promotes the proliferation of ISCs.

### Hedgehog signaling pathway

The Hedgehog (Hh) signaling pathway is a paracrine in the strict sense. In humans, three Hh ligands are expressed: Sonic Hedgehog (Shh), Indian Hedgehog (Ihh), and Desert Hedgehog (Dhh). Ihh is one of the important ligands of the Hedgehog pathway and maintains the stability of the ISCs’ niche (49). Ihh is secreted by IECs and acts on mesenchymal cells. Mesenchymal cells produce signaling factors that negatively regulate the proliferation of ISCs. Moreover, the BMP pathway plays a synergistic role in this process (50).

### Intestinal vascular endothelial cell injury

Endothelial cell damage mediated by IR is also a pathophysiology of Re. Radiation intestinal epithelial injury is the main cause of acute radiation enteropathy, while chronic radiation enteropathy is caused by vascular endothelial injury. Vascular endothelial cells are sensitive to IR (51). Irradiation of the vascular system can rupture blood vessels and induce a pro-inflammatory response (52). After endothelial injury, subendothelial extracellular matrix (ECM) components are exposed to platelets, which initiate the hemostatic mechanism by forming the thrombus (53). Due to the excessive secretion of the von Willebrand factor (vWF) by damaged endothelial cells, the coagulation cascade is over-activated, resulting in vascular occlusion (54). This can then lead to hyperemia or hemorrhage at the site of injury due to increased vascular permeability. This is why the irradiated gut has a poor blood supply (55). The progression of Re can be improved by reducing IR damage to the vascular endothelium (56).

Shao et al. (57) reported that ferulic acid (FA) can reduce the oxidative damage of radiation to endothelial cells. The thrombomodulin (THBD) pathway may be an important mechanism of FA against radiation injury. Endothelial acid sphingomyelinase can catalyze ceramide production, which leads to endothelial cell apoptosis. Rotolo et al. (58) reported that IR can lead to the activation of the endothelial acid sphingomyelinase, thus initiating cell apoptosis. The 2A2 is an anti-ceramide IgM, which can prevent endothelial cell apoptosis in the lamina propria of the small intestine. In addition, the 2A2 can promote the recovery of ISCs.

Endothelial thrombomodulin (TM) is a multi-domain transmembrane receptor protein with anti-inflammatory, cytoprotective, antifibrinolytic, antioxidant, and anticoagulant functions (59). The high expression of TGF-β means endothelial damage, permeability destruction, and endothelial dysfunction (60, 61). TM suppresses the TGF-β signaling pathway by inhibiting extracellular signal-regulated kinase (ERK) activation (62). Pathak et al. (63) proved that TM treatment significantly ameliorated Re.

Yan et al. (64) reported that ionizing radiation can lead to reduced intestinal blood supply, resulting in intestinal ischemia and induced Re. BH4 can improve intestinal blood perfusion, which is mainly achieved through the Gh1/BH4/eNOS pathway. After intestinal exposure to ionizing radiation, guanosine triphosphate (GTP) cyclic hydrolase 1 (Gch1) will decrease, and BH4 is regulated by Gch1 and will decrease with the decrease of Gch1. Endothelial nitric oxide synthase (eNOS) must be completely saturated with BH4 to synthesize nitric oxide (NO). NO can relax vascular smooth muscle and maintain blood perfusion. Exogenous BH4 supplementation significantly improved the function of intestinal endothelial cells and intestinal blood perfusion, and alleviated pathological injury, thus preventing radiation enteritis. They further studied that ligustilide (LIG) can also prevent Re through the Gch1/BH4/eNOS pathway (65). The mechanism of action of LIG is to ameliorate the decrease of Gch1 protein level, thereby increasing BH4 and NO content. Compared with the control group, the LIG group significantly increased the length of intestinal villi. In addition, pretreatment with LIG improved weight loss and diarrhea caused by radiation. These results can reflect that LIG pretreatment has a positive effect on the prevention of Re.

### Intestinal microflora

The human intestinal microflora contains 10^14^ species of resident microorganisms that live in the human intestinal tract with bacteria, viruses, fungi, and protozoa (66). Some studies have shown that intestinal microflora helps break down other indigestible polysaccharides in our diet, regulates the storage of calories extracted from our diet in fat cells, metabolizes foreign substances including carcinogens, regulates intestinal epithelial cell turnover, and educates the immune system how to respond to external stimuli (67). Metabolites produced by microbial communities play an important role in maintaining homeostasis and internal environment stability (68). The destruction of intestinal microflora is closely related to Re (69).

Touchefeu et al. believed that there are significant changes in intestinal microflora in patients receiving radiotherapy, with the most common being a decrease in the Clostridium cluster XIVa, Bifidobacterium, *Faecalibacterium prausnitzii*, and an increase in Enterobacteriaceae. These modifications may lead to mucositis, bacteremia, and diarrhea (70). Johnson et al. (71) believed that pseudo-intestinal obstruction and bacterial overgrowth may occur after abdominal radiotherapy. Impaired motor function is one of the causes of gastrointestinal colonization of Gram-negative bacilli. The results of Crawford and Gordon’ showed that compared with ordinary mice, germ-free mice received a lethal dose of total body irradiation, the survival rate was significantly higher, and the survival time was significantly longer (67).

Urolithin A (UroA) is a metabolite of intestinal microflora. Zhang et al. (72) found that UroA at 2 mg/kg significantly improved the survival and regeneration of intestinal structure and intestinal epithelium in rats exposed to ionizing radiation. In addition, UroA can regulate the structure of intestinal microbiome. IR can further increase the abundance of *Escherichia shigella*, Proteobacteria, Alphaproteobacteria, and Erysipelotrichaceae. The proliferation of these microflora leads to the destruction of the structure and function of the intestinal barrier and promotes intestinal inflammation, but UroA can reverse this result and prevent the occurrence of Re.

## Conclusion

Cancer therapy continues to improve, but radiation therapy remains an important part of cancer treatment, and Re is an inevitable side effect of radiotherapy. Interventions for Re often determine the efficacy of radiation therapy in patients. The occurrence of Re is usually not determined by unilateral factors, and the complex interaction between intestinal epithelium, ISCs, capillary endothelium, and luminal bacteria is considered to be the basis of Re pathogenesis. Resveratrol, TZC01, HKST, etc. could reduce Re in animal experiments, which needs to be confirmed by clinical trials.

## Author contributions

JF and BL drafted the manuscript, and MF, TN, FG and BT participated in the data review and collection for the study. XD conceived the study and participated in its design and coordination. All authors read and approved the final manuscript.

## Funding

This work was financially supported by the NHC Key Laboratory of Nuclear Technology Medical Transformation (Mianyang Central Hospital, grant no. 2022HYX008) and Natural Science Foundation of Sichuan Province (grant no. 2022NSFSC0849).

## Conflict of interest

The authors declare that the research was conducted in the absence of any commercial or financial relationships that could be construed as a potential conflict of interest.

## Publisher’s note

All claims expressed in this article are solely those of the authors and do not necessarily represent those of their affiliated organizations, or those of the publisher, the editors and the reviewers. Any product that may be evaluated in this article, or claim that may be made by its manufacturer, is not guaranteed or endorsed by the publisher.
